# Effects of dietary sodium butyrate supplementation on fat metabolism in lamb adipose and liver tissues

**DOI:** 10.5713/ab.24.0919

**Published:** 2025-06-24

**Authors:** Yue Zhang, Hongbo Qu, Yueying Guo, Mirco Corazzin, Min Zhang, Ting Liu, Lin Su, Lihua Zhao, Lina Sun, Ye Jin

**Affiliations:** 1 College of Food Science and Engineering, Inner Mongolia Agricultural University, Hohhot, China; 2 Department of Agricultural, Food, Environmental and Animal Sciences, University of Udine, Udine, Italy

**Keywords:** Adipose-liver Axis, Fat Distribution, Lamb Adipose Tissue, Nutritional Regulation, Sodium Butyrate

## Abstract

**Objective:**

Sodium butyrate (SB) is a potentially useful feed additive; however, its effects on lipid metabolism in adipose and liver tissues of lambs are still not fully explored. This study systematically examined the effects and underlying mechanisms of dietary SB supplementation on lipid metabolism in lamb adipose and liver tissues from an adipose-blood-liver perspective.

**Methods:**

Twelve 3-month-old male lambs (22.37±2.05 kg) were randomly divided into a control group and an SB group. We measured the adipose tissue cellular morphology and lipid metabolism-related indices in both adipose and liver tissues.

**Results:**

The results indicated that SB significantly reduces abdominal and perirenal adipose tissue mass, as well as the average area and diameter of adipocytes (p<0.05). Dietary supplementation with SB activated adenosine 5′-monophosphate-activated protein kinase α1 (AMPKα1) in lamb adipose tissue, resulting in upregulated mRNA expression of hormone-sensitive triglyceride lipase (HSL) and downregulated mRNA expression of sterol regulatory element-binding protein 1 and fatty acid synthase (p<0.05). Simultaneously, adiponectin secretion and receptor expression in adipose tissue, as well as serum adiponectin levels, were significantly elevated (p<0.05). Moreover, dietary supplementation with SB increased the levels of tricarboxylic acid cycle metabolites in lamb liver, including oxaloacetate, citrate, cis-aconitate, and succinate (p<0.05), while simultaneously activating the liver AMPKα1 signaling pathway. These changes led to upregulated HSL, platelet glycoprotein 4, and long-chain acyl-CoA synthetase mRNA expression (p<0.05), thereby enhancing liver fatty acid metabolism.

**Conclusion:**

In summary, dietary supplementation with SB alters adiponectin levels in lambs, activates the AMPK signaling pathway, promotes adipose tissue lipolysis, and regulates liver lipid metabolism. The findings provide valuable insights into the use of SB for managing lamb body fat reserves and offer a robust basis for further research in animal bioscience.

## INTRODUCTION

Adipose tissue is considered the largest and most efficient energy storage organ in mammals, it adjusts the size of adipocytes through lipolysis and lipogenesis, adapting to changes in organismal energy requirements [1]. Adipocytes participate in physiological processes such as growth, development, and immune regulation, influencing the activities of other organs through hormones, cytokines, and fatty acids [2]. Adipose tissue secretes hormones such as adiponectin and leptin, which regulate the metabolic activities of peripheral organs [3]. However, research indicates that adiponectin and leptin do not operate independently, they participate in adipose metabolism alongside hormones like insulin and catecholamine, forming a novel regulatory network with systemic impacts [4]. This network may be providing new mechanisms for interactions between adipose tissue and other organs.

The liver serves as a central metabolic organ in animals, responsible for the synthesis, metabolism, storage, and redistribution of lipids, being crucial to maintaining and regulating lipid metabolism within the body and engaging in interactions with diverse organs including the brain, intestines, adipose tissue, and skeletal muscle [5]. Research indicates that the liver is one of the key target organs for the action of adiponectin secreted by adipose tissue, adiponectin can modulate the lipid metabolism of the liver through adiponectin receptors (AdipoR1/2) [6]. Meanwhile, adiponectin modulates the fatty acid metabolism of the liver through the adenosine 5′-monophosphate (AMP)-activated protein kinase (AMPK), and influences the level of enzymes that are crucial for fatty acid synthesis and oxidation [7]. This indicates that under physiological conditions, the adipose-liver axis represents a critical pathway regulating lipid metabolism.

Adipose tissue plays a vital role in the energy balance of animals. As animals mature, adipose tissue accumulates and deposits in different fat depots within the body. However, the accumulation of abdominal adipose tissue (AAT) and perirenal adipose tissue (PAT) poses challenges for processors, as these tissues become part of the final product, but consumers may not like this, which can lead to a reduction in the commercial value of the product [8]. Therefore, regulating fat distribution in carcasses has become a focal point of global livestock research. In recent years, altering feeding strategies is regarded as an effective way to regulate the pathways of fat synthesis and breakdown in animals and to optimize fat deposition. Producers have endeavoured to utilize dietary nutrition approaches as a breakthrough, modulating fat metabolism to reduce carcass fat deposition.

Sodium butyrate (SB) is a widely used feed supplement. It has been established that SB positively development of the rumen in ruminant animals while also acting as a key regulator in maintaining intestinal barrier function and inflammation [9]. Study have confirmed that SB can regulate the function of peripheral tissues (liver, adipose tissue) to influence systemic energy homeostasis in animals [10]. In particular, SB can enhance mitochondrial oxidative phosphorylation by activating the AMPK pathway mediated by adiponectin, promoting the expression of fatty acid oxidation enzymes. However, the impact of dietary supplementation with SB on lipid metabolism in lamb adipose and liver tissues remains unexplored.

Therefore, this study hypothesizes that dietary supplementation with SB can regulate the secretion of the key adipokine adiponectin via the AMPK signaling pathway, thereby modulating the adipose-liver axis lipid metabolism in lambs, promoting white fat decomposition, and optimizing carcass fat reserves. This study investigated the mechanisms of dietary SB supplementation on lipid metabolism in lamb fat and liver tissues, clarifying the key regulatory roles of lipid metabolism-related hormones in this process. This study provides a theoretical basis for the future application of SB in feed to improve fat reserves in lamb carcasses.

## MATERIALS AND METHODS

### Animals experiment

All experimental procedures involving animals were approved by the Animal Ethics Committee of Inner Mongolia Agricultural University, under the approval number NND2024065. Twelve male lambs, with similar weights (22.37±2.05 kg), were chosen and randomly assigned to two groups, each containing of 6 animals. The lambs remained with their mothers and nursed for 90 days. On the 90th day, the lambs were separated from their mothers and raised individually in a pen. The size of each pen was 4 m^2^. The lambs were fed either a basic diet (Supplement 1; CON group) or the basic diet with the addition of 1% SB group. To ensure full intake of the SB, 100 g of the basal diet was first thoroughly mixed with the daily amount of SB and offered individually to each lamb in the SB group. Once this portion was completely consumed, the remaining basal diet was then provided. The basic diet met the recommended nutritional requirements for lamb [11]. The experimental period lasted 90 days, during which all animals consumed their feed without refusal, and water was provided ad libitum throughout the study.

### Samples collection

After a fasting of 12 h, all the lambs were slaughtered at an abattoir located 1.5 km from the farm. Prior to slaughter (9:00 a.m.), blood samples (15 mL) were collected from the jugular vein of each lamb using centrifuge tubes containing EDTA as an anticoagulant and stored at 4°C. After slaughter, excess moisture on the surface of AAT from the rectus abdominis area and PAT around the left kidney was blotted dry using absorbent paper. The samples were weighed using an electronic scale with a 1,000 g capacity. The adipose tissue mass was recorded. The adipose tissue samples were processed by dividing them into two portions: the first portion was cut into 1 cm^3^ cubes and placed in 5 mL centrifuge tubes containing 4% (v/v) paraformaldehyde solution for adipocyte morphology analysis, while the second portion was cut into 1 cm×1 cm× 1.5 cm rectangular blocks, placed in 2 mL cryovials, and stored at −80°C for enzyme activity, adipokines, and gene expression analysis. Liver tissue samples (1 cm×1 cm×1.5 cm) were also collected, with three pieces placed in each 2 mL cryovial and stored at −80°C for the determination of liver metabolites, enzyme activity, and gene expression. All samples from each lamb were collected within 10 min post-slaughter.

### Measurement of growth performance

Before the start and at the end of the experiment, each lamb was weighed individually. The daily feed intake for each animal was recorded to calculate the average daily feed intake (ADFI) during the experiment was calculated. Additionally, the average daily gain (ADG) and feed conversion ratio (FCR) were determined.

### Measurement of serum metabolites

Blood samples were centrifuged at 3,000×g for 15 min at 4°C, and the supernatant was collected. Triglycerides (TG, H201), total cholesterol (TC, H202), high-density lipoprotein cholesterol (HDL-C, H203), low-density lipoprotein cholesterol (LDL-C, H207), and free fatty acids (NEFA, H444) levels were measured using a Hitachi biochemical analyzer (HITACHI 3110). The serum insulin (MM-8004301), epinephrine (MM-256802), leptin (MM-3723001), and adiponectin (MM-8105801) concentrations were determined using a sheep-specific and double antibody sandwich ELISA kit (Ovis, Jiangsu Meimian Industrial).

### Measurement of liver metabolites

A 0.05 g sample was weighed and placed into a 2 mL centrifuge tube, with the weight of each sample recorded. Immediately, 500 μL of pre-chilled (−20°C) 70% methanol/water extraction solution was added. The samples were then ground using a cryogenic grinder (Jingxin MIX-200) at a frequency of 70 Hz for 3 min, followed by vortexing for 3 min under the condition of 2,500×g and centrifuged at 12,000×g for 10 min at 4°C and 300 μL of the supernatant were carefully transferred to a 1.5 mL centrifuge tube, kept at −20°C for 30 min, and centrifuged again at 12,000×g for 10 min at 4°C. Finally, 200 μL of the supernatant was collected and filtered through a protein precipitation plate (Thermo Fisher Scientific) for analysis. The detection of all target metabolites (Acetyl-CoA, ATP, AMP, cAMP, lactate, malic acid, oxaloacetate, citric acid,cis-aconitic acid, and succinic acid) were carried out using the AB Sciex QTRAP 6500 LC-MS/MS platform.

### Adipocytes morphology measurement

The samples were dehydrated with a tissue dehydrator (HistoCore Pear I; Leica) and embedded in paraffin by a paraffin embedding machine (HistoCore Arcadia H+C, Leica). The paraffin blocks containing the tissue were then sliced into 5 μm sections using a rotary microtome (HistoCore AUTOCUT; Leica). Then, three sections per sample were stained with hematoxylin and eosin. Three random areas of each section were observed under a digital microscope (LEICA DN4000 B LED; Leica) equipped with a 10× objective lens. The number, average diameter, and average area of adipocytes were quantified using ImageJ 1.x software, and the mean values from the nine measurements (three areas per section× three sections per sample) were used for further statistical analysis.

### Concentrations of fat metabolism related enzymes, adiponectin and leptin

0.5 g of liver and adipose tissue samples (AAT and PAT) were weighed, and 9 times the volume of 0.9% sodium chloride aqueous solution was added. The samples were ground using a cryogenic grinder (Jingxin MIX-200) at a frequency of 70 Hz for 3 min, followed by centrifugation at 1,500×g for 15 min at 4°C. The supernatant from the tissue was then collected. The concentrations of AMP-activated protein kinase α1 (AMPKα1, MM-8117001), acetyl-CoA carboxylase 1 (ACC1, MM-8116301), carnitine palmitoyltransferase 1A (CPT1A, MM-202001), carnitine palmitoyltransferase 1B (CPT1B, MM-202001), hormone sensitive lipase (HSL, MM-8103101), lipoprotein lipase (LPL, MM-204801), fatty acid synthase (FASN, MM-8116901), and stearoyl-CoA desaturase (SCD, MM-8103801) in the liver and adipose tissue were determined using ELISA kits (Ovis, Jiangsu Meimian Industrial). Additionally, concentrations of adiponectin (MM-8105801) and leptin (MM-3723001) in adipose tissue were determined using ELISA kits (Ovis, Jiangsu Meimian Industrial). All the ELISA kits were specifically designed for lamb.

### Expression of genes related to lipid metabolism

The samples were ground in liquid nitrogen using a sterile mortar and pestle, and RNA was extracted using RNAiso Plus reagent (No. 9109; Takara). The concentration and purity of each RNA sample were measured using a Nanodrop spectrophotometer (Thermo Fisher Scientific). The RIN numbers of all the RNA in this study ranged from 8.2–10. Then, the RNA was reverse transcribed into cDNA using PrimeScript RT reagent (No. RR047; Takara). Then and the expression levels of target genes were quantified using SYBR Green real-time PCR quantification kit. Each sample was analyzed in quintuplicate (five technical replicates), and the mean Ct value was used for the calculation of relative gene expression levels. The primer sequences are listed in Supplement 2. Gene-specific primers were synthesized by Shanghai Sangon Biotech. The primers efficiency was verified using the standard curve obtained by amplification of dilution of the pooled cDNA. Relative expression levels were determined using the 2^−ΔΔCt^ method to normalize target gene mRNA to actin beta (ACTB).

### Statistical analysis

The metabolism products in the liver were quantitatively analyzed using Multiquant 3.0.3 software (Sciex). Experimental data were initially processed using Excel (2016) and subjected to statistical analysis using SPSS 26.0. For growth performance data, initial body weight was included as a covariate in the model. Diet treatment was specified as a fixed effect. Normality was tested using the Shapiro–Wilk method, and homogeneity of variance was assessed using Levene’s test. Data conforming to both assumptions were analysed via t-tests, while the Mann-Whitney U test was applied for non-normal or heteroscedastic data. Graphs were plotted using GraphPad Prism 9.0. Results in the tables were expressed as mean values and standard errors of the mean (SEM), and results in the conclusions were supplemented by mean values and standard deviation, both for the CON group vs SB group. Differences were considered highly significant at p<0.01, statistically significant at p<0.05 and a trend towards significant at 0.05≤p<0.10.

## RESULTS

### Performance

Table 1 presented the effect of SB on the growth performance of lambs from the beginning to the end of the experiment, and the results show that SB had no differential effect on the initial and final weight of lambs (p>0.05). However, the addition of SB significantly increased the ADG and decreased the FCR (p<0.05).

### Adipose tissue characteristics

Table 2 showed the effect of SB addition to the diet on adipose tissue characteristics of lambs. Compared to the CON group, the SB group exhibited a significant reduction in the mass, mean area of adipocyte and diameter of adipocyte in AAT and PAT (p<0.05). In the SB group, the number of PAT significantly increased (228.17±6.014 *vs* 254.36±5.194, p<0.05), while the number of AAT showed no significant change (p> 0.05) (Supplement 3).

### Fat metabolism-related enzymes of the adipose tissue

Table 3 indicated that in AAT, the levels of FASN was noticeably reduced in lambs from the SB group (7.15±0.304 nmol/L) compared to the CON group (8.88±0.597 nmol/L, p<0.05). Furthermore, SB group showed a tendential higher and lower levels of AMPKα1 and ACC1 (0.05≤p<0.10). Within the PAT, the level of AMPKα1 was significantly higher (266.79±4.931 pg/mL *vs* 329.29±7.715 pg/mL, p<0.05), and the level of ACC1 was lower in lambs from the SB group (224.85±7.427 pmol/L *vs* 197.69±9.770 pmol/L, p<0.05). SB group had higher CPT1B and HSL values than CON group, but the differences did not achieve statistical significance (p>0.05).

### Fat metabolism gene expression in the adipose tissue

Illustrated by Table 4, compared to the CON group, lambs from the SB group exhibited significantly increased mRNA expression levels of AMPKα1 (0.90±0.101 *vs* 1.14±0.09, p<0.05) and CPT1B (1.08±0.334 *vs* 2.53±0.264, p<0.05) in the PAT, whereas no significant differences were observed in AAT (p>0.05). Interestingly, the expression level of ACC1 mRNA was significantly lower in both AAT (1.14±0.127 *vs* 0.89± 0.091, p<0.05) and PAT (4.44±1.138 *vs* 1.43±0.059, p<0.05) of lambs from the SB group. Furthermore, lambs from the SB group demonstrated a significant increase in the expression levels of HSL mRNA in both AAT (0.71±0.062 *vs* 1.25±0.319, p<0.05) and PAT (0.81±0.093 *vs* 1.16±0.017, p<0.05), while the mRNA expression levels of SREBF1 and FASN were significantly reduced (p<0.05).

### The level of adiponectin and leptin in the adipose tissue

As showed in Table 5, the adiponectin in both AAT (139.96± 0.268 μg/L *vs* 171.02±0.231 μg/L, p<0.05) and PAT (185.23± 1.124 μg/L *vs* 206.31±2.687 μg/L, p<0.05) of lambs from the SB group significantly increased. The level of leptin was not significantly different (p>0.05).

### Serum lipid metabolites

As indicated in Table 6, the SB group exhibited significantly higher the serum levels of adiponectin (42.14±3.013 μg/L *vs* 66.77±3.018 μg/L, p<0.05) and epinephrine (105.06±2.031 ng/L *vs* 138.46±1.431 ng/L, p<0.05) in lambs, while significantly decreasing the insulin level in the serum (84.06±6.046 mU/L *vs* 74.63±4.540 mU/L, p<0.05). The SB group had a numerically higher leptin concentration than CON group, but the differences was not statistically significant (p>0.05). SB group showed lower concentrations of TG (0.55±0.108 mmol/L *vs* 0.40±0.071 mmol/L, p<0.05), TC (1.56±0.177 mmol/L *vs* 1.32±0.137 mmol/L, p<0.05), and LDL-C (0.82±0.120 mmol/L *vs* 0.50±0.101 mmol/L, p<0.05), and higher NEFA (0.08±0.029 mmol/L *vs* 0.13±0.027 mmol/L, p<0.05) concentration than CON group.

### Liver metabolites

As indicated in Table 7, the content of acetyl-CoA in the liver of lambs from the SB group exhibited a significant increased (255.97±25.129 ng/g *vs* 357.19±8.872 ng/g, p<0.05), accompanied by a significant decrease in ATP (3,660.25±47.321 ng/g *vs* 2,044.82±95.937 ng/g, p<0.05) and cAMP (39.30±3.844 ng/g *vs* 22.53±2.132 ng/g, p<0.05) levels. SB group had higher AMP/ATP ratio than CON group (p<0.05). The content of representative metabolites of tricarboxylic acid cycle (TCA) metabolism, oxaloacetate (977.26±67.307 ng/g *vs* 4,064.62± 96.032 ng/g, p<0.05), citric acid (250.47±13.703 ng/g *vs* 338.77±16.112 ng/g, p<0.05), cis-aconitic acid (6.97±0.153 ng/g *vs* 9.40±0.567 ng/g, p<0.05), and succinate acid (1,687.96±166.055 ng/g *vs* 4,198.17±34.380 ng/g, p<0.05), was significantly increased in the liver of lambs in the SB group.

### Enzymes activity and genes expression related to lipid metabolism in the liver

Table 8 illustrated the impact of dietary SB supplementation on liver lipid metabolism-related enzymes in lambs. The content of AMPKα1 (370.56±16.233 pg/mL *vs* 419.46±10.486 pg/mL, p<0.05), CPT1A (1,455.94±67.938 ng/L *vs* 2,020.00± 72.920 ng/L, p<0.05), and HSL (532.01±8.148 μg/L *vs* 568.40± 8.678 μg/L, p<0.05) in the liver of lambs from the SB group was significantly higher than the CON group, while the content of ACC1 (378.86±7.847 pmol/L *vs* 268.06±8.520 pmol/L, p<0.05), and SCD (561.42±30.998 ng/L *vs* 466.69±14.773 ng/L, p<0.05) was significantly lower than the CON group. The results from Table 9 demonstrated that, the mRNA expression levels of AMPKα (0.89±0.074 *vs* 2.01±0.204, p<0.05), HSL (1.42±0.260 *vs* 1.68±0.188, p<0.05), LPL (1.23±0.209 *vs* 2.45± 0.228, p<0.05), platelet glycoprotein 4 (CD36, 1.07±0.068 *vs* 2.03±0.394, p<0.05), and long chain acyl-CoA synthetase1 (ACSL1, 1.16±0.251 *vs* 1.64±0.308, p<0.05) in the liver from the SB group were significantly upregulated, while the mRNA expression levels of ACC1 (0.73±0.122 *vs* 0.47±0.121, p<0.05), FASN (2.23±0.513 *vs* 1.22±0.271, p<0.05), and SCD (0.77±0.122 *vs* 0.43±0.112, p<0.05) were significantly downregulated.

### Expression of adipose metabolism-related hormone receptor genes in adipose tissue

The results in Table 10 show that the expression levels of AdiopR1 mRNA in both AAT (0.68±0.154 *vs* 1.07±0.221, p<0.05) and PAT (1.01±0.020 *vs* 1.30±0.198, p<0.05) of lambs from the SB group were significantly higher than those in the CON group, at the same time, the expression level of AdiopR2 mRNA in PAT (1.11±0.153 *vs* 1.67±0.235, p<0.05) was significantly increase. However, dietary supplementation with SB did not significantly affect adipose tissue ADRB mRNA and LEPR mRNA expression in lambs (p>0.05).

## DISCUSSION

From the perspective of adipose tissue cell morphology, this study found that dietary supplementation with SB decreased the average diameter and area of adipocytes in the AAT and PAT of lambs. This may be the primary reason for the reduction in the fat mass of the AAT and PAT due to SB [12]. This indicates that SB may induce a tendency for the breakdown of AAT and PAT in lambs. It has been suggested that SB can affect the size and number of lipid droplets, reduce the proliferation rate of adipocytes, and decrease fat accumulation [13]. Study suggest that SB promotes fat breakdown by activating AMPK and inducing the phosphorylation of its downstream target gene, acetyl-CoA carboxylase [14]. However, this conclusion has not been previously confirmed in lamb fat metabolism. To explore the impact of SB on lamb fat metabolism, the level of lipid metabolism-related enzymes and the mRNA expression of genes in AAT and PAT were measured. We found that dietary supplementation with SB appeared to activate the AMPKα1-ACC1 signalling pathway in adipose tissue, which is believed to regulate fatty acid oxidation. Furthermore, the mRNA expression results in adipose tissue showed that SB supplementation activated the expression of AMPKα1 mRNA in PAT in lambs, mediating fatty acid oxidation through the AMPKα1-ACC1-CPT1B pathway. While SB cause a numerically increased, in the level of the key enzyme HSL involved in fat breakdown in lamb adipose tissue, the expression of HSL mRNA was significantly upregulated in both the AAT and PAT of lambs, leading to a notable reduction in triglyceride deposition in adipocytes. Study performed in mice have demonstrated that supplementation with butyrate can reverse the decrease of HSL and LPL in the adipose tissue of obese individuals, resulting in increased fat hydrolysis and a decreased content of lipids [15]. These findings suggest that alterations in the level of lipolytic enzymes and the expression of gene patterns may constitute pivotal factors in SB improving the fat deposition of lambs.

The processes of lipolysis and synthesis in adipose tissue are critical determinants of fat deposition, playing a central role in regulating organismal energy metabolism and homeostasis. This process is jointly regulated by adipose tissue-secreted adiponectin, leptin and hormones released by peripheral tissues, forming a metabolic regulatory network [6]. Insulin is a crucial regulator of adipose tissue, inhibiting the activity of HSL in adipose tissue and reducing the mobilization rate of NEFA from adipose tissue, thereby suppressing lipolysis, while adrenaline serves as an effective regulator of lipolysis, inhibiting fat synthesis and stimulating lipolysis [16]. In this study, supplementation with SB led to a decrease in the level of insulin and an increase in the level of adrenaline in lamb serum, while the level of NEFA in lamb plasma increased. This could be attributed to SB supplementation altering the concentrations of various plasma hormones, accelerating the release of fatty acids, and increasing the rate of lipolysis. Additionally, leptin and adiponectin, as important adipokines secreted by adipose tissue, also play pivotal roles in metabolic regulatory network. The level of adiponectin in the body is typically negatively correlated with the mass of adipose tissue, indicating that the lower the mass of adipose tissue, the higher the level of adiponectin [17]. Adipose tissue produces adiponectin and releases it into the bloodstream, exerting a critical role in lipid regulation in the blood. In agreement with the previously reported papers, in the present study the supplementation with SB stimulated the production of adiponectin in both the AAT and PAT of lambs, consequently increasing the levels of adiponectin in the blood. The hydrolysis products of the adiponectin protein can stimulate fatty acid oxidation and reduce blood triglycerides, with SB possibly serving as the triggering factor for this series of reactions [18]. In the results of our study, the levels of TG, TC, and LDL-C in the blood of lambs decreased. Similar to adiponectin, leptin is another plasma protein primarily synthesized and secreted by mature adipocytes, regulating energy expenditure and food intake by binding to specific receptors in the hypothalamus [19]. The concentration of leptin correlates positively with body fat content. In this study, supplementation with SB did not significantly affect the ability of lamb adipose tissue to secrete leptin, nor did it alter leptin levels in the blood, indicating that the regulation of lamb adipose tissue by SB may be primarily mediated by adiponectin. In addition to its mediation through adiponectin, SB itself can induce multiple effects through the bloodstream. It promotes cholesterol metabolism by converting more cholesterol into bile acids, thereby reducing the cholesterol secretion of the liver and lowering the concentration of TC and LDL-C in the blood [20].

In ruminants, butyrate can be metabolized at the liver level. Specifically, butyrate can form acetyl-CoA by AMP or act on acetyl-CoA in β-oxidation. This is consistent with the results of this study, where dietary supplementation with SB led to an increase in the level of acetyl-CoA in the liver of lamb. Acetyl-CoA can be converted to citrate, generating adenosine triphosphate through complete oxidation in the TCA or shuttling outside the mitochondria [21]. We observed that supplementation with SB increased the levels of citric acid, oxaloacetate, cis-aconitic acid, and succinate acid in the TCA pathway of the liver. This suggests that SB may promote the TCA cycle to some extent. Furthermore, SB can regulate the mitochondrial dynamics and efficiency of the liver, leading to increased phosphorylation of the AMPKα1-ACC1 pathway [22]. This indicates that SB may influence the lipid metabolism of the liver through an AMPK-dependent mechanism. Our study results demonstrated a significant increase in the levels of AMPKα1, CPT1A, and HSL enzyme in the lamb liver following supplementation with SB, while the levels of ACC1 and SCD were significantly decreased. ACC, acting as the rate-limiting enzyme in fatty acid synthesis in the liver, catalyzes the conversion of acetyl-CoA to malonyl-CoA (an inhibitor of CPT1A, a key regulator of fatty acid oxidation) [23]. These findings confirm that butyrate can improve the lipid metabolism of the liver by reducing fat formation (ACC) or enhancing intracellular fat breakdown (AMPKα1, CPT1A, and HSL) enzyme. In this study, the AMPK signalling pathway in lamb liver was activated by SB, leading to the occurrence of AMPK cascade effects in the liver. Specifically, the expression of AMPK downstream factors including ACC1, FASN, and SCD mRNA was downregulated, consistent with the levels of enzymatic in the liver. This indicates that SB may modulate the transcriptional regulation of AMPK, a crucial factor in fatty acid synthesis and oxidation processes in the liver [24]. Activation of AMPK resulted in increased expression of the fatty acid transporters CD36 and ACSL1 mRNA in the liver. CD36 plays a pivotal role in lipid metabolism by facilitating the translocation of fatty acids within hepatocytes, thereby facilitating fatty acid oxidation and breakdown. Similarly, ACSL1 facilitates the entry of fatty acids into mitochondria for oxidation [25]. This indicates that the activation of AMPK mediated by SB regulates the lipid metabolism of the liver in multiple aspects, including fatty acid synthesis, oxidation, and transport. Surprisingly, adiponectin, may serve as crucial mediators in this process. Studies suggest that the impact of SB on the lipid metabolism of the liver may partly depend on the final control of AMPK in the liver, leading to increased fatty acid oxidation and inhibition of triglyceride accumulation [26]. The adiponectin receptor AdipoR2 is primarily expressed in the liver, and its stimulation results in phosphorylation and subsequent inhibition of acetyl-CoA carboxylase, reducing the amount of malonyl-CoA, thereby alleviating its inhibitory effect on CPT1A and facilitating the entry of long-chain acyl-CoA into mitochondria for β-oxidation [27].

It is noteworthy that adiponectin and leptin, which are self-secreted by adipose tissue, and hormones released by peripheral tissues have autoregulatory effects on the lipid metabolism of adipose tissue. In vitro studies have shown that both subtypes of adiponectin receptors can mediate AMPK phosphorylation through adiponectin binding, activating fatty acid oxidation. In this study, supplementation with SB increased adiponectin secretion in the adipose tissue of lamb. Additionally, the expression of adiponectin receptors in adipose tissue was upregulated, with a significant increase in AdipoR1 and AdipoR2 mRNA expression in PAT, while only AdipoR1 mRNA expression increased in AAT. This difference may be due to variations in the responsiveness of different adipose tissue depots to hormone-receptor interactions. Adiponectin is predominantly expressed in PAT, while leptin is primarily sourced from AAT [28]. However, in this study, SB did not significantly affect the secretion of leptin or the expression pattern of LEPR mRNA in adipose tissue. Therefore, it is likely that SB primarily regulates the lipid metabolism of lamb through adiponectin mediation. Unexpectedly, we observed an increase in adrenaline levels in the blood following SB supplementation, accompanied by an upregulation of ADRB receptor expression in the adipose tissue. This suggests that SB can alter the expression of ADRB in adipose tissue [29]. It is believed that activated ADRB not only phosphorylates HSL to initiate lipid degradation, significantly enhancing the ability of adipocytes to metabolize lipids, but also inhibits fatty acid synthesis and esterification of fatty acids into triglycerides [30]. Furthermore, it has been demonstrated that SB activates the ADRB-AMPKα signalling pathway to promote the β-oxidation of fat and reduce lipid accumulation in 3T3-L1 adipocytes, aligning with the activation of the AMPKα signalling pathway in adipose tissue mentioned earlier, regulating the AMPK-ACC1-CPT1B pathway associated with fat oxidation.

## CONCLUSION

In summary, dietary supplementation with SB promotes the breakdown of AAT and PAT in lambs. This may be due to SB altering the levels of adiponectin, thereby activating the AMPK signalling pathway in adipose tissue to facilitate fatty acid oxidation and degradation. Additionally, this process leads to modify regulation of blood lipid metabolites in lambs, accelerates the TCA cycle, and synchronously activates the AMPK signalling pathway in the liver, regulating the lipid metabolism of the liver through various mechanisms including fatty acid oxidation, synthesis, and transport. Consequently, dietary supplementation with SB can regulate fat distribution by modulating lipid metabolism in lamb adipose and liver tissues.

## Figures and Tables

**Table 1:** Effect of sodium butyrate addition on the growth performance of lambs

Parameter	Treatment		SEM	p-value

	CON	SB		
Initial BW (kg)	23.61	21.67	0.612	0.116
Final BW (kg)	53.56	55.50	0.667	0.573
ADG (kg/d)	0.32	0.38	0.010	0.047
ADFI (kg/d)	1.41	1.40	0.187	0.556
Carcass weight (kg)	25.96	27.20	0.470	0.433
FCR (kg/kg)	4.29	3.74	0.110	0.039

CON, control group; SB, sodium butyrate group; SEM, standard error of the mean; BW, body weight; ADG, average daily gain; ADFI, average daily feed intake; FCR, feed conversion ratio.

**Table 2:** Effect of dietary sodium butyrate addition on abdominal and perirenal adipose tissue characteristics of lambs

Tissue site	Parameter	Treatment		SEM	p-value

		CON	SB		
AAT	Mass (kg)	0.45	0.36	0.02	0.025
	Number of adipocytes	275.00	271.09	6.46	0.778
	Mean area of adipocyte (um^2^)	3,182.20	2,750.44	102.75	0.040
	Mean diameter of adipocyte (um)	77.37	70.83	1.30	0.019
PAT	Mass (kg)	0.77	0.58	0.05	0.034
	Number of adipocytes	228.17	254.36	4.35	0.001
	Mean area of adipocyte (um^2^)	3,991.84	3,545.57	81.68	0.003
	Mean diameter of adipocyte (um)	92.46	88.40	0.87	0.023

CON, control group; SB, sodium butyrate group; SEM, standard error of the mean; AAT, abdominal adipose tissue; PAT, perirenal adipose tissue.

**Table 3:** Effect of sodium butyrate addition on the key enzymes of lipid metabolism of abdominal and perirenal adipose tissue in lambs

Tissue site	Parameter	Treatment		SEM	p-value

		CON	SB		
AAT	AMPKα1 (pg/mL)	231.67	311.43	22.51	0.056
	ACC1 (pmol/L)	228.70	213.43	4.46	0.075
	CPT1B (ng/L)	1,407.50	1,428.85	50.13	0.854
	HSL (μg/L)	364.74	378.27	12.02	0.624
	LPL (ng/L)	1,922.50	1,925.63	53.76	0.980
	FASN (nmol/L)	8.88	7.15	0.39	0.007
	SCD (ng/L)	332.50	437.40	24.72	0.004
PAT	AMPKα1 (pg/mL)	266.79	329.29	15.52	0.033
	ACC1 (pmol/L)	224.85	197.69	6.29	0.010
	CPT1B (ng/L)	960.10	1199.69	76.11	0.119
	HSL (μg/L)	404.46	415.00	13.89	0.748
	LPL (ng/L)	1,869.38	1,826.56	30.11	0.532
	FASN (nmol/L)	8.56	7.71	0.35	0.294
	SCD (ng/L)	452.11	445.15	6.86	0.660

CON, control group; SB, sodium butyrate group; SEM, standard error of the mean; AAT, abdominal adipose tissue; AMPKα1, protein kinase AMP-activated catalytic subunit alpha 1; ACC1, acetyl CoA carboxylase 1; CPT1B, carnitine palmitoyltransferase 1B; HSL, hormone sensitive triglyceride lipase; LPL, lipoprteinlipase; FASN, fatty acid synthase; SCD, stearoyl-CoA desaturase; PAT, perirenal adipose tissue.

**Table 4:** Effect of dietary supplementation with sodium butyrate on fat metabolism related gene expression in abdominal and perirenal adipose tissue of lambs

Tissue site	Parameter	Treatment		SEM	p-value

		CON	SB		
AAT	AMPKα1	1.04	1.22	0.09	0.320
	ACC1	1.14	0.89	0.06	0.018
	CPT1B	0.59	0.85	0.09	0.171
	HSL	0.71	1.25	0.14	0.035
	LPL	1.94	1.64	0.09	0.116
	SREBF1	1.23	0.69	0.13	0.020
	FASN	1.76	1.51	0.07	0.044
	SCD	0.52	0.63	0.03	0.106
PAT	AMPKα1	0.90	1.14	0.06	0.050
	ACC1	4.44	1.43	0.03	0.004
	CPT1B	1.08	2.53	0.31	0.002
	HSL	0.81	1.16	0.09	0.024
	LPL	1.68	1.43	0.09	0.261
	SREBF1	0.77	0.52	0.07	0.009
	FASN	2.89	2.08	0.20	0.032
	SCD	0.94	0.87	0.14	0.824

CON, control group; SB, sodium butyrate group; SEM, standard error of the mean; AAT, abdominal adipose tissue; AMPKα1, protein kinase AMP-activated catalytic subunit alpha 1; ACC1, acetyl CoA carboxylase 1; CPT1B, carnitine palmitoyltransferase 1B; HSL, hormone sensitive triglyceride lipase; LPL, lipoprteinlipase; SREBF1, sterol regulatory element-binding protein 1; FASN, fatty acid synthase; SCD, stearoyl-CoA desaturase; PAT, perirenal adipose tissue.

**Table 5:** Effect of dietary supplementation with sodium butyrate on adiponectin and leptin in abdominal and perirenal adipose tissue of lambs

Tissue site	Parameter	Treatment		SEM	p-value

		CON	SB		
AAT	Leptin (ng/L)	3.72	3.17	0.19	0.170
	Adiponectin (μg/L)	139.96	171.02	9.21	0.026
PAT	Leptin (ng/L)	3.30	3.20	0.51	0.877
	Adiponectin (μg/L)	185.23	206.31	5.81	0.043

CON, control group; SB, sodium butyrate group; SEM, standard error of the mean; AAT, abdominal adipose tissue; PAT, perirenal adipose tissue.

**Table 6:** Effect of dietary supplementation with sodium butyrate on serum metabolites and secretions in lambs

Parameter	Treatment		SEM	p-value

	CON	SB		
Triglyceride (mmol/L)	0.55	0.40	0.04	0.022
Total cholesterol (mmol/L)	1.56	1.32	0.06	0.035
HDL-C (mmol/L)	0.55	0.57	0.02	0.412
LDL-C (mmol/L)	0.82	0.50	0.06	0.002
NEFA (mmol/L)	0.08	0.13	0.01	0.006
Insulin (mU/L)	84.06	74.63	2.37	0.037
Adiponectin (μg/L)	42.14	66.77	6.64	0.033
Leptin (ng/L)	3,212.08	3,437.08	100.95	0.299
Epinephrine (ng/L)	105.06	138.46	8.59	0.013

CON, control group; SB, sodium butyrate group; SEM, standard error of the mean; HDL-C, high-density lipoprotein cholesterol; LDL-C, low-density lipoprotein cholesterol; NEFA, free fatty acid.

**Table 7:** Effect of dietary supplementation with sodium butyrate on the liver metabolites in lambs

Parameter	Treatment		SEM	p-value

	CON	SB		
Acetyl-CoA (ng/g)	255.97	357.19	23.04	0.008
ATP (ng/g)	3,660.25	2,044.82	328.52	0.005
AMP (ng/g)	20,475.18	26,034.41	1,910.40	0.162
AMP/ATP (ng/g)	4.68	14.88	2.10	0.001
cAMP (ng/g)	39.30	22.53	3.46	0.007
Lactate (ng/g)	8,444.88	9,004.68	269.37	0.326
Malic acid (ng/g)	66,145.69	71,815.63	2,453.23	0.271
Oxaloacetate (ng/g)	977.26	4,064.62	924.27	0.036
Citric acid (ng/g)	250.47	338.77	18.33	0.004
Cis-aconitic acid (ng/g)	6.97	9.40	0.63	0.047
Succinic acid (ng/g)	1,687.96	4,198.17	499.73	0.002

CON, control group; SB, sodium butyrate group; SEM, standard error of the mean; ATP, adenosine triphosphate; AMP, adenosine monophosphate; cAMP, cyclic adenosine monophosphate.

**Table 8:** Effect of dietary sodium butyrate addition on lipid metabolism-related enzymes in the liver of lambs

Parameter	Treatment		SEM	p-value

	CON	SB		
AMPKα1 (pg/mL)	370.56	419.46	12.96	0.046
ACC1 (pmol/L)	378.86	268.06	24.81	0.005
CPT1A (ng/L)	1,455.94	2,020.00	9.65	0.035
HSL (μg/L)	532.01	568.40	171.19	0.049
LPL (ng/L)	2,436.56	2,567.81	55.50	0.267
FASN (nmol/L)	13.86	13.99	0.29	0.861
SCD (ng/L)	561.42	466.69	20.68	0.003

CON, control group; SB, sodium butyrate group; SEM, standard error of the mean; AMPKα1, protein kinase AMP-activated catalytic subunit alpha 1; ACC1, acetyl CoA carboxylase 1; CPT1A, carnitine palmitoyltransferase 1A; HSL, hormone sensitive triglyceride lipase; LPL, lipoprteinlipase; FASN, fatty acid synthase; SCD, stearoyl-CoA desaturase.

**Table 9:** Effect of dietary sodium butyrate supplementation on the expression of genes related to liver lipid metabolism in lambs

Parameter	Treatment		SEM	p-value

	CON	SB		
AMPKα1	0.89	2.01	0.14	<0.001
ACC1	0.73	0.47	0.04	0.001
CPT1A	0.93	1.14	0.07	0.134
HSL	1.42	1.68	0.06	0.025
LPL	1.23	2.45	0.21	<0.001
FASN	2.23	1.22	0.17	<0.001
SCD	0.77	0.43	0.05	<0.001
ACSL1	1.16	1.64	0.10	0.009
CD36	1.07	2.03	0.16	<0.001

CON, control group; SB, sodium butyrate group; SEM, standard error of the mean; AMPKα1, protein kinase AMP-activated catalytic subunit alpha 1; ACC1, acetyl CoA carboxylase 1; CPT1A, carnitine palmitoyltransferase 1A; HSL, hormone sensitive triglyceride lipase; LPL, lipoprteinlipase; FASN, fatty acid synthase; SCD, stearoyl-CoA desaturase; ACSL1, long-chain acyl-CoA synthase1; CD36, platelet glycoprotein 4.

**Table 10:** Effect of dietary sodium butyrate supplementation on hormone receptor gene expression in adipose tissue of lambs

Tissue site	Parameter	Treatment		SEM	p-value

		CON	SB		
AAT	AdiopR1	0.68	1.07	0.10	0.037
	AdiopR2	0.67	0.75	0.04	0.342
	LEPR	0.52	0.44	0.06	0.591
	ADRB	0.57	0.93	0.13	0.177
PAT	AdiopR1	1.01	1.30	0.07	0.032
	AdiopR2	1.11	1.67	0.11	0.002
	LEPR	0.22	0.19	0.02	0.504
	ADRB	2.03	2.12	0.22	0.850

CON, control group; SB, sodium butyrate group; SEM, standard error of the mean; AAT, abdominal adipose tissue; AdiopR1, adiponectin receptor 1; AdiopR2, adiponectin receptor 2; LEPR, leptin receptor; ADRB, adrenoceptor beta 2; PAT, perirenal adipose tissue.

## SUPPLEMENTARY MATERIAL

**Supplement 1**. The ingredient composition and nutritional level of the basic diet.

**Supplement 2**. The primer names, sequences and product sizes.

**Supplement 3**. Effect of dietary supplementation with sodium butyrate on adipocyte morphology in lambs.
