# Supplementary material for: Effects of dietary sodium butyrate supplementation on fat metabolism in lamb adipose and liver tissues
Source: Anim Biosci. 2025 Jun 24;38(12):2679–89. doi: 10.5713/ab.24.0919 (PMC12580741; doi:10.5713/ab.24.0919)
Supplement: Supplementary file 1 [file ab-24-0919-Supplementary-1.pdf]

Supplement 1. The ingredient composition and nutritional level of the basic diet.

Ingredient, %	
Corn	65.00
soybean meal	12.00
Wheat bran	18.00
Premix[1]	3.00
Salt	1.00
Chemical composition, %	
Crude protein	14.00
Crude fiber	20.00
Crude ash	12.00
Ca	0.40
Total P	0.30
Moisture	14.00
Lysine	0.30

[1] One kg of premix contained the nutrients as following: vitamin A 150 KIU, vitamin3 30 KIU, vitamin E 800 mg, Fe ($FeSO_4$) 600 mg, Cu ($CuSO_4$) 150 mg, Zn ($ZnSO_4$) 1200mg, Mn ($MnSO_4$) 500 mg, Se (Na_2SeO_3) 5 mg, I ($Ca(IO_3)_2$) 12 mg.
