# Supplementary material for: Effects of dietary sodium butyrate supplementation on fat metabolism in lamb adipose and liver tissues
Source: Anim Biosci. 2025 Jun 24;38(12):2679–89. doi: 10.5713/ab.24.0919 (PMC12580741; doi:10.5713/ab.24.0919)
Supplement: Supplementary file 2 [file ab-24-0919-Supplementary-2.pdf]

Supplement 2. The primer names, sequences and product sizes.

Gene Name [1]	Sequence (5'-3')	Product size(bp)
ACTB	F: CTCACGGAGCGTGGCTACA<br>R: GCCATCTCCTGCTCGAGGTC	107
AMPKα1	F: TCCGAAGTATTGATGATGA<br>R: ACAGATGAGGTAAGAGAAG	154
ACC1	F: GACACATCACATCCGTCCTCT<br>R: GTCCATCACCACAGCCTTCAT	188
CPT1A	F: CCTTCCCATTCCBCACTTT<br>R: CGGTCTCTGTTCTGCCCTCT	171
CPT1B	F: TGTTCAACACCACTCGCATC<br>R: CTCGTAGAGCCACAGCTTGA	116
HSL	F: CTTTCGCACCAGCCACAAC<br>R: CTCGTCGCCCTCAAAGAAGA	136
LPL	F: TCATCGTGGTGGACTGGCT<br>R: CATCCGCCATCCAGTTCATA	111
FASN	F: GTGTGGTACAGCCCCTCAAG<br>R: ACGCACCTGAATGACCACTT	110
SCD	F: GAGTACCGCTGGCACATCAA<br>R: CTAAGACGGCAGCCTTGGAT	103
CD36	F: TGTGTTTGGAGGGATTCT<br>R: CCTTGGCTAACGAACTCTG	242
ACSL1	F: GCCATCACCTACATCATCAACAA<br>R: ACACTTCTTGCCTCGTTCCA	171
SREBF1	F: CGCAAAGCCATCGACTACATC<br>R: TGAGCTTCTGGTTGCTGTGCT	52
ADIPOR1	F: GCTGAAGTGAGAGGAAGAGTC<br>R: GAGGGAATGGAGTTTATTGCC	118

ADIPOR2	F: GGCAACATCTGGACACATC<br>R: CTGGAGACCCCTTCTGAG	200
LEPR	F: GAAGCCTGATCCACCATTAG<br>R: CATCCAATCTCTTGCTCCTC	239
ADRB	F: ACCTGCCCCTGGTAGTCATG<br>R: TAGCGTCTTGAGGGCTTTGTG<br>R: GGACGCACTTGTTGTTGTGAATGAC	200

[1] ACTB= actin beta; AMPKα1= protein kinase AMP-activated catalytic subunit alpha 1; ACC1 = acetyl CoA carboxylase 1; CPT1A = carnitine palmitoyltransferase 1A; CPT1B = carnitine palmitoyltransferase 1B; HSL = hormone sensitive triglyceride lipase; LPL = lipoprteinlipase; FASN = fatty acid synthase; SCD = stearoyl-CoA desaturase; CD36 = platelet glycoprotein 4; ACSL1 = long chain acyl-CoA synthetase1; SREBF = sterol regulatory element bingding transcription factor 1; ADIOPR1 = adiponectin receptor 1; ADIOPR2 = adiponectin receptor 2; LEPR = leptin receptor; ADRB = adrenoceptor beta.
