# Supplementary material for: Effects of dietary sodium butyrate supplementation on fat metabolism in lamb adipose and liver tissues
Source: Anim Biosci. 2025 Jun 24;38(12):2679–89. doi: 10.5713/ab.24.0919 (PMC12580741; doi:10.5713/ab.24.0919)
Supplement: Supplementary file 3 [file ab-24-0919-Supplementary-3.pdf]

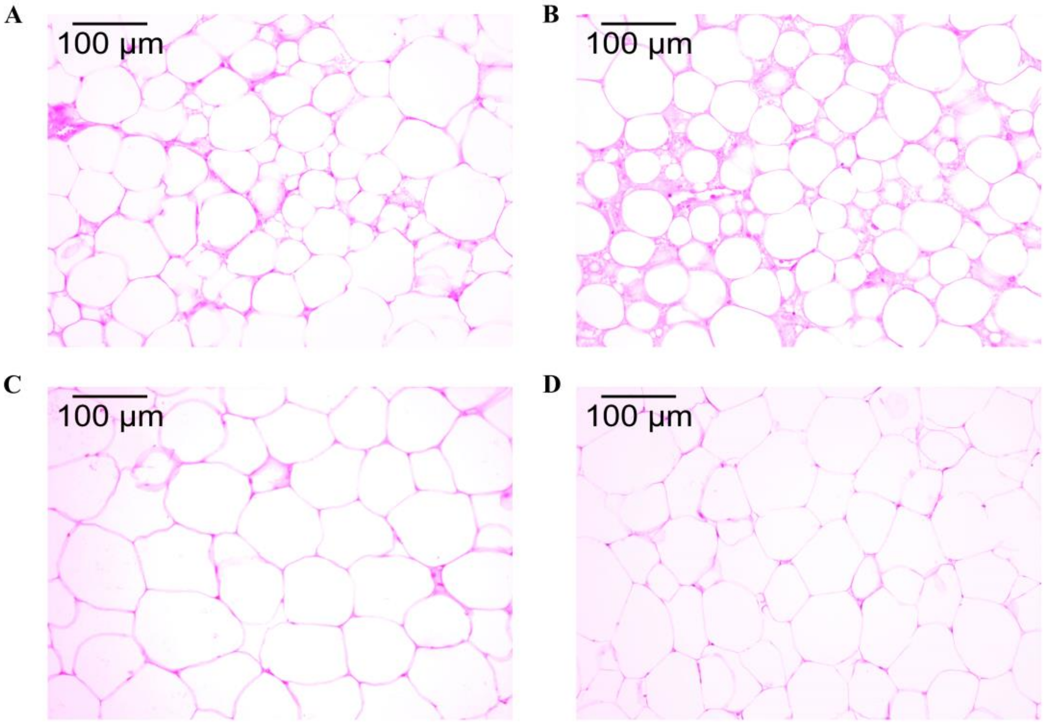

Supplement 3. Effect of dietary supplementation with sodium butyrate on adipocyte morphology in lambs. (A), (B) Representative H&E staining of the abdominal adipose tissue in control group and sodium butyrate group. (C), (D) Representative H&E staining of the perirenal adipose tissue in control group and sodium butyrate group.
